# Methicillin-resistant *Staphylococcus aureus* among urban rodents, house shrews, and patients in Guangzhou, Southern China

**DOI:** 10.1186/s12917-019-2012-8

**Published:** 2019-07-25

**Authors:** Jing Ge, Xue-shan Zhong, Yi-quan Xiong, Min Qiu, Shu-ting Huo, Xue-jiao Chen, Yun Mo, Ming-ji Cheng, Qing Chen

**Affiliations:** Guangdong Provincial Key Laboratory of Tropical Disease Research, School of Public Health, Southern Medical University, Guangzhou, China

**Keywords:** Urban rodent, House shrew, Methicillin-resistant *Staphylococcus aureus*, Antimicrobial resistance, Multilocus-sequence typing

## Abstract

**Background:**

The transmission of methicillin-resistant *Staphylococcus aureus* (MRSA) between humans and animals has been identified in a number of countries. In this study, MRSA in urban rodents and shrews in a community was investigated. Further, comparisons of MRSA isolates from rodents, shrews, and humans were conducted to evaluate the relationships of these isolates from different origins.

**Results:**

Between 2015 and 2016, 397 oropharynx samples from 212 rodents and 185 shrews, and 8 MRSA isolates from hospital patients were collected. Twelve MRSA were isolated from the small mammals (3.0, 95%CI: 1.3–4.7%), including 11 isolates from rodents and one from a shrew. Three MRSA isolates from *Rattus norvegicus* were PVL-positive, and seven isolates were IEC-negative (one from *Suncus murinus*, five from *Rattus norvegicus*, and one from a patient). The *spa* type, MLST, and antimicrobial resistance patterns showed that the MRSA retrieved from rodents and shrews are likely related to human strains.

**Conclusion:**

MRSA derived from rodent shares similar antimicrobial resistance and molecular characteristics to those from humans, suggesting that urban rodents may play as maintenance host or vectors for MRSA which is important to human health.

## Background

*Staphylococcus aureus* (*S. aureus*) is an important opportunistic pathogen, which causes a wide range of infections in humans and animals [1]. *S. aureus* has the capacity to rapidly develop resistance to most antibiotics used clinically [2]. For example, in 1961, 2 years after the clinical introduction of methicillin in the United Kingdom, the first methicillin-resistant *S. aureus* (MRSA) strain was identified [3]. Since then, MRSA infections have increased worldwide. In 2014 the World Health Organization reported that for all-cause mortality, MRSA had a Relative Risk (RR) of 1.61 compared to methicillin-susceptible *S. aureus* (MSSA) infections (95% Confidence Interval, CI: 1.43–1.82) [4]. Initially, MRSA was primarily hospital associated but in the late 1990s MRSA emerged as a community-associated infection (CA-MRSA) [5]. CA-MRSA caused serious infections in younger and healthier individuals and was unusually virulent with a marked capacity to disseminate throughout the community [6].

*S. aureus* methicillin resistance is due to the acquisition of the *mecA* gene that encodes a penicillin-binding protein (PBP 2a) with a low affinity for β-lactams [7]. A number of investigations have identified animals as potential reservoirs of *mecA*-containing staphylococci [8, 9]. Multiple animal-associated staphylococci have also been identified as the probable origin of methicillin resistance conferred by *mecA* [10, 11]. These reservoirs include companion animals [12, 13], livestock [14], and wildlife [15]. Transmission of MRSA between animals and humans, in both directions, has been well documented [16, 17].

In addition to domestic animals and livestock, urban wildlife such as rodents and shrews can also serve as reservoirs for zoonotic bacterial disease [18–20]. The first such report was livestock-associated MRSA (LA-MRSA) in rats living on pig farms in the Netherlands [21] in 2009, and more recently in urban Norway rats in Canada [22]. The Norway rat (*Rattus norvegicus*) [23] and the Asian house shrew (*Suncus murinus*) [24] are predominantly commensal animals in urban areas within regions of China. However, to the best of our knowledge, no information is available regarding the prevalence, antimicrobial resistance, or genetic characteristics of MRSA sampled from urban rodents in China. Additionally, there was no report on description of MRSA in shrews throughout the world.

The aims of this study were to determine the carriage rate, drug-resistance, and genetic characteristics of *S. aureus* and MRSA in urban rodents and shrews in Guangzhou City, Southern China. Further, in order to evaluate the relationships of MRSA isolates among these animals and humans, comparisons of molecular characterization and antibiotic susceptibility were conducted.

## Results

### Animal trapping and bacterial isolation

In total, 397 individual animals, including 212 rodents (197 *Rattus norvegicus* and 15 *Rattus tanezumi*) and 185 shrews (185 *Suncus murinus*), were captured between June 2015 and May 2016 (Table 1)*.* They were captured near the garbage bin, on the lawn, and near house building in relation to human habitation.

**Table 1:** Distribution of *S. aureus* and MRSA carriage in urban rodents and shrews trapped between 2015 and 2016 in Guangzhou, Southern China

Species	No. of captures	*S. aureus* no. (%)	MRSA no. (%)
Rodents	212	87/212 (41.0)	11/212 (5.2)
*Rattus norvegicus*	197	83/197 (42.1)	11/197 (5.6)
*Rattus tanezumi*	15	4/15 (26.7)	0/15 (0)
Shrews			
*Suncus murinus*	185	14/185 (7.6)	1/185 (0.5)
Total	397	101/397 (25.4)	12/397 (3.0)

*S. aureus Staphylococcus aureus*;

*MRSA* Methicillin-resistant *Staphylococcus aureus*

Of the 397 animals trapped, *S. aureus* was isolated from 101 (25.4, 95% CI: 21.1–29.7%), of those 87 isolates were derived from rodents and 14 from shrews. *S. aureus* carriage rates in rodents (41.0, 95% CI: 34.4–47.7%) were higher than in shrews (7.6, 95% CI: 3.7–11.4%) (χ^2^ = 58.3, *p* < 0.001) (Table 1). Of the 101 *S. aureus* isolates, 12 were *mecA-*positive MRSA (3.0, 95% CI: 1.3–4.7%), including 11 from *Rattus norvegicus* and one from *Suncus murinus*. The MRSA carriage rate was 5.2% (95% CI: 2.2–8.2%) in rodents and 0.5% in shrews.

**Fig. 1:**
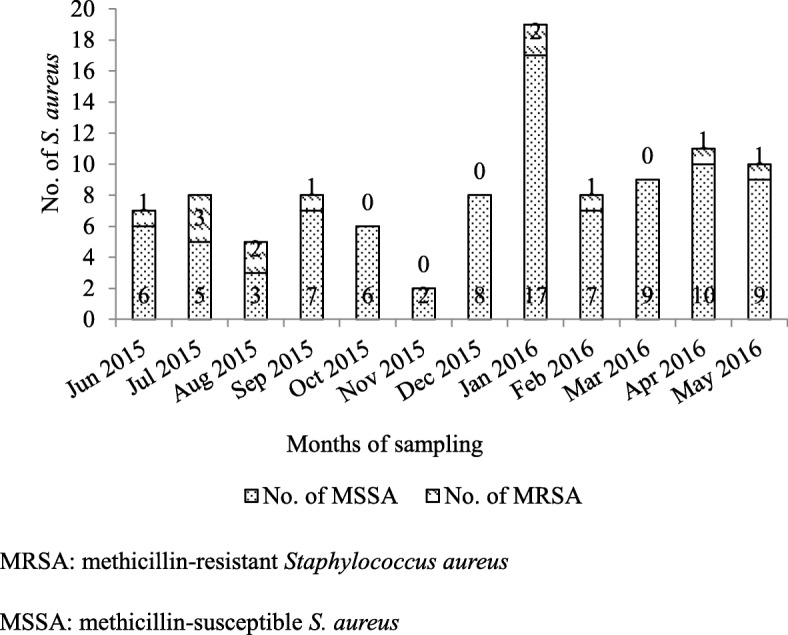
Distribution of MRSA and MSSA carriage in urban rodents and shrews by month between 2015 and 2016 in Guangzhou, Southern China. MRSA: methicillin-resistant *Staphylococcus aureus*. MSSA: methicillin-susceptible *S. aureus*

There was significant difference in seasonal carriage rates for *S. aureus*: summer (June to August, 17.2, 95% CI: 10.3–24.2%), fall (September to November, 12.3, 95% CI: 6.6–18.0%), winter (December to February, 46.1, 95% CI: 34.6–57.5%), and spring (March to May, 40.0, 95% CI: 28.7–51.3%) (χ^2^ = 41.336, *p* < 0.001). There was no difference in MRSA seasonal carriage rates: summer (5.2%), fall (0.8%), winter (3.9%), and spring (2.7%) (χ^2^ = 4.335, *p* = 0.227) (Fig. 1).

### Antimicrobial susceptibility

All 101 *S. aureus* isolates were susceptible to minocycline, gentamicin, vancomycin, and trimethoprim-sulfamethoxazole. Of 89 MSSA isolates, the resistance rate to penicillin was the highest (50.6%), followed by azithromycin (20.2%), tetracycline (10.1%), clindamycin (3.4%), linezolid (1.1%), rifampin (1.1%) chloramphenicol (1.1%), and ciprofloxacin (1.1%). Four MSSA isolates showed intermediate resistance to clindamycin (*n* = 3) and nitrofurantoin (*n* = 1) (data are not shown).

Sixteen MDRSA isolates were detected among the *S. aureus* isolates. The proportion of MDRSA to *S. aureus* isolated from urban rodents and shrews was 16.1% (14/87) and 14.3% (2/14), respectively. Among these MDRSA isolates, four were MSSA (one from shrew and three from rodents).

The MRSA isolate derived from the shrew was susceptible to most antibiotics tested with the exception of penicillin, cefoxitin, and tetracycline. Among the 11 rodent MRSA isolates, resistance rates to penicillin and cefoxitin were highest (7/11), followed by azithromycin (3/11), clindamycin (3/11), tetracycline (3/11), rifampin (2/11), and ciprofloxacin (1/11). One rodent MRSA isolate showed intermediate susceptibility to tetracycline (Table 2).

**Table 2:** Antimicrobial-resistant patterns of 20 MRSA isolates from rodents, shrews, and patients between 2015 and 2016 in Guangzhou, Southern China

ID	Host	Antimicrobial resistance													
		AZM	CM	CFX	P	SXT	LZD	MH	TE	RD	C	CIP	CN	F	VA^c^
185	SM	S	S	R	R	S	S	S	I	S	S	S	S	S	≤1
213	RN	S	S	R	R	S	S	S	R	R	S	R	S	S	≤1
215	RN	S	S	R	R	S	S	S	S	S	S	S	S	S	≤1
239	RN	R	R	R	R	S	S	S	R	S	S	S	S	S	≤1
257	RN	R	R	S	R	S	S	S	S	S	S	S	S	S	≤1
263	RN	S	S	R	R	S	S	S	S	R	S	S	S	S	≤1
329	RN	S	S	R	R	S	S	S	S	S	S	S	S	S	≤1
65	RN	S	S	S	S	S	S	S	S	S	S	S	S	S	≤1
466	RN	S	S	S	S	S	S	S	S	S	S	S	S	S	≤1
484	RN	S	S	R	S	S	S	S	S	S	S	S	S	S	≤1
533	RN	R	R	R	R	S	S	S	I	S	S	S	S	S	≤1
564	RN	S	S	S	S	S	S	S	R	S	S	S	S	S	≤1
18	PA	R	S	R	R	S	S	S	S	I	S	S	S	S	≤1
07	PA	R	S	R	R	S	S	S	R	R	S	R	R	S	≤1
06	PA	R	R	R	R	R	S	I	R	S	S	R	R	S	≤1
73	PA	R	R	R	R	S	S	S	I	I	R	S	S	S	≤2
67	PA	R	I	S	R	S	S	S	R	S	S	R	S	S	≤1
418	PA	S	S	S	S	S	S	S	S	S	S	S	S	S	≤1
95	PA	R	R	R	R	S	S	I	R	S	S	R	R	S	≤2
76	PA	R	R	R	R	S	S	S	S	S	S	S	S	S	≤2

*MRSA* Methicillin-resistant *Staphylococcus aureus*

*SM Suncus murinus*, *RN Rattus norvegicus*, *PA* Patient, *AZM* azithromycin, CM clindamycin, *CFX* cefoxitin, *P* penicillin, *SXT* trimethoprim/sulfamethoxazole, *LZD* linezolid, *MH* minocycline, *TE* tetracycline, *VA* vancomycin, *RD* rifampin, *C* chloramphenical, *CIP* ciprofloxacin, *CN* gentamicin, *F* nitrofurantoin, *R* resistant, *I* intermediate, *S* susceptible

^c^MICs for vancomycin were determined by the agar dilution method

Drug resistance profiles for the patient MRSA isolates (*n* = 8) were similar to the profiles of isolates collected from animals in this study. Antibiotic-resistance rates were highest for β-lactams (penicillin/cefoxitin) and azithromycin (7/8). The resistant rates to clindamycin, tetracycline, ciprofloxacin, and gentamycin were 50%(4/8). One MRSA isolate was resistant to trimethoprim/sulfamethoxazole. Two isolates exhibited intermediate susceptibility to minocycline. All eight MRSA isolates were susceptible to linezolid, nitrofurantoin, and vancomycin (Table 2).

### Molecular characterization of MRSA

**Fig. 2:**
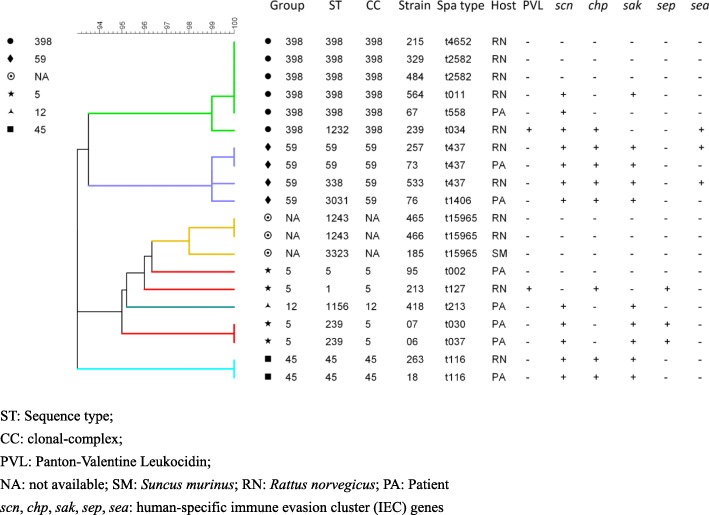
Molecular characteristics of 20 MRSA isolates from rodents, shrews, and patients between 2015 and 2016 in Guangzhou, Southern China. ST: Sequence type; CC: clonal-complex; PVL: Panton-Valentine Leukocidin; NA: not available; SM: *Suncus murinus*; RN: *Rattus norvegicus*; PA: Patient, *scn*, *chp*, *sak*, *sep*, *sea*: human-specific immune evasion cluster (IEC) genes

Among 11 MRSA isolates retrieved from rodents, 8 *spa* types were identified including t437 (*n* = 2), t2582 (*n* = 2), t127 (*n* = 1), t4652 (*n* = 1), t034 (*n* = 1), t116 (*n* = 1), t011 (*n* = 1), and t15965 (*n* = 2), which t15965 was a new *spa* type not observed previously. One MRSA isolate from shrew also belonged to t15965. Eight different *spa* types were detected among eight MRSA isolates from patients including t116, t030, t037, t437, t558, t213, t002, and t1406. The *spa* type t116 and t437 were found in both rodents and humans (Fig. 2).

After MLST analysis, seven sequence types (STs) were detected among 11 rodent MRSA isolates. The dominant type was ST398 (*n* = 4), followed by ST1243 (*n* = 2), ST59 (*n* = 1), ST45 (*n* = 1), ST1232 (*n* = 1), ST1 (*n* = 1), and ST338 (*n* = 1). A new type, ST3323, was identified from the shrew MRSA isolate. Seven STs were detected among the eight human MRSA isolates including ST45, ST239, ST59, ST398, ST1156, ST5, and ST3031. ST45, ST59, and ST398 were found in both rodents and humans (Fig. 2).

By eBURST analysis, 20 MRSA isolates (derived from the small animals and from humans) were divided into 6 groups based on Clonal Complexes (CCs) (Fig. 2). Five CCs were available, including CC5, CC398, CC59, CC45 and CC12. CC5 included ST1-t127, ST239-t030, ST239-t037, and ST5-t002. CC398 included ST398-t4652, ST1232-t034, ST398-t2582, ST398-t011, and ST398-t558. CC59 included ST59-t437, ST338-t437, and ST3031-t1406. CC45 included ST45-t116, and CC12 included ST1156-t213. Clonal Complexes of three isolates were not available (ST1243-t15965 and ST3323-t15965) (Fig. 2).

Among 101 *S. aureus* isolates from small animals only three MRSA isolates from *Rattus norvegicus* were PVL-positive, including CC398-ST1232-t034, CC59-ST59-t437, and CC59-ST338-t437. The PVL gene was not detected among eight MRSA isolates from patients (Fig. 2).

Among 11 MRSA isolates from rodents, five isolates were IEC-negative, including two isolates with a new type (ST1243-t15965) and another three isolates belonging to CC398 (ST398-t4652, ST1232-t034, and ST398-t2582). The shrew MRSA isolate belonged to a new type (ST3323-t15965) and was IEC-negative. Among eight patient MRSA isolates only one isolate (CC5-ST5-t002) was IEC-negative (Fig. 2).

## Discussion

This study is the first to characterize *S. aureus* and MRSA in urban rodents in China. To the best of our knowledge, it is also the first report of carriage rates and bacterial characteristics of MRSA in house shrews worldwide. The most striking finding is that urban rodents and house shrews carry a diverse MRSA strains, including those found in humans and domestic animals. The house shrew, a small mole-like mammal, originated from the Indian subcontinent and are now found from southern Asia and Afghanistan to the Malay Archipelago and southern Japan [25]. As commensal animals, urban rodents and house shrews are commonly found near human households. The proximity to human is important in the transmission of pathogens.

The prevalence of *S. aureus* among urban rodents in this study was 25.4% (101/397, 95% CI: 21.1–29.7%), with a prevalence 41.0% (95% CI: 34.4–47.7%) in rodents and 7.6% (95% CI: 3.7–11.4%) in shrews. In total, the MRSA carriage rate among these animals was 3.0% (95% CI: 1.3–4.7%). In rodents and shrews, the MRSA carriage rate was 5.2% (95% CI: 2.2–8.2%) and 0.5%, respectively. The reported *S. aureus* carriage rate in rodents varies from 7.1% (43/608) [15] to 41.86% (18/43) [21]. The highest prevalence of MRSA was 11.6% (5/43) reported in rats living on pig farms in the Netherlands [21], followed by 3.5% (22/637) in urban rats in Canada [22]. These differences in carriage rates may be due to geographic differences, sample source, or sample size [26]. The carriage rates of *S. aureus* and MRSA in rodents and shrews could also be influenced by season. MRSA carriage among wild urban Norway rats was higher in the winter and spring as described in previous study [22]. A similar seasonality was noted for MRSA infection rates in humans in previous research, with CA-MRSA peaking in late summer and HA-MRSA peaking in the winter [27]. In this study, rodents and shrews caught in the summer and fall had a lower-carriage rate of *S. aureus* than in the winter and spring. While, no seasonal variation was observed among MRSA carriage rates. The seasonality of MRSA carriage rate among urban rodents and house shrews in Guangzhou, Southern China needs further research.

Antibiotic resistance reported here differs from a previous study looking at MRSA in urban-dwelling *Rattus norvegicus* [22]. In this study resistance to penicillin was 63.64% versus 100% (in the previously reported study), cefoxintin 63.64% versus 100%, rifampin 18.18% versus 9.09%, clindamycin 27.27% versus 0, and ciprofloxacin 9.09% versus 0. These differences may relate to geographic location and/or the genotypes of these MRSA isolates [28]. All MRSA isolates from the small animals were susceptible to trimethoprim/sulfamethoxazole, minocycline, gentamicin, and vancomycin, which is in agreement with a previous evaluation of human and animal MRSA isolates in China [29–31].

To differentiate human- from livestock-associated *S. aureus* isolates, clonal complexes (CCs) and resistance patterns have been used by other researchers [32, 33]. Absence of the human-specific IEC genes can be used to identify livestock-associated *S. aureus* [34], as those genes were lost with the evolution of LA-MRSA from its human-adapted ancestor [32]. As such, CCs, resistance patterns, and the presence of PVL or IEC genes (such as *scn*, *chp*, *sak*, *sea*, and *sep*) were used in this study to identify the potential origin of MRSA isolates.Current *S. aureus* typing methods include *spa* typing, MLST, *SCCmec* typing, pulsed-field gel electrophoresis (PFGE), and multilocus variable-number tandem-repeat (VNTR) analysis (MLVA) [35]. However, no single-typing method is ideal [36, 37]. Based on international-standard nomenclature and flexibility, *spa* typing and MLST were used in this study.

MRSA CC398, which is also referred to as LA-MRSA, is the most frequent CC detected in this study, even though *S. aureus* CC9 has been identified as the main livestock-associated clone in most Asian countries [38, 39]. The CC398-t034 clone has been shown to be dominant among MSSA isolates from rodents [40]. MRSA ST398 has been detected in *Rattus norvegicus* from an inner-city neighborhood [22], suggesting that urban rats could be an important reservoir for *S. aureus* CC398 isolates. Generally, LA-MRSA ST398 is characteristically resistant to tetracycline, and lacks both PVL and IEC genes [41]. It is noteworthy that none of the five MRSA CC398 isolates in this study showed this typical pattern. Three MRSA CC398 isolates that lacked IEC genes were susceptible to tetracycline and were PVL-negative, suggesting that these isolates may have an animal origin. In contrast, the remaining two MRSA CC398 isolates were tetracycline resistant, IEC-positive, with one carrying the PVL gene, suggesting that these two isolates may have a human origin. Interestingly, one of patient-derived MRSA isolates shared the same pattern as that of rat isolates, PVL-negative, susceptible to tetracycline, and IEC-positive. In point of fact, LA-MRSA CC398 originated as a MSSA in humans and jumped to livestock by the acquisition of methicillin and tetracycline resistance and by the loss of phage-carried, human-virulence genes [32]. Recently, CC398 has been split into two distinct lineages, those that are of human origin and those that are livestock adapted [42].

*S. aureus* clones previously identified mainly in humans in Asian countries were also isolated from both *Rattus norvegicus* and patients in this study. These clones included MRSA CC59 (ST59-t437 and ST338-t437), the major CA-MRSA clones in China, and common HA-MRSA, CC5 (ST1-t127) and CC45 (ST45-t116) [38]. The molecular characterization of these MRSA clones conforms to the profiles of CA-MRSA and HA-MRSA [5]. More work needs to be done, such as long-read sequencing, to assess potential transmission events of CA-MRSA and/or HA-MRSA from humans to rodents or vice-versa.

In this study, three MRSA isolates were found to belong to a new-*spa* type (t15965) not previously identified. These isolates lacked PVL and IEC genes, suggesting derivation from animals. However, these isolates were from different MLST (ST3323 and ST1243) and had varying antimicrobial susceptibility patterns. Two of these were retrieved from *Rattus norvegicus*, belonging to ST1243, and susceptible to all antibiotics tested. The remaining one was retrieved from *Suncus murinus*, belonging to ST3323, and resistant to cefoxitin and penicillin, with intermediate resistance to tetracycline. Further analysis is required to confirm these resistance patterns, their associated host reservoir species, and their transmissibility.

## Conclusion

This study is the first to report the carriage and characteristics of MRSA in urban house shrews. It is also the first to characterize MRSA isolates from urban rodents in China. The *spa* type, MLST, and antimicrobial resistance patterns suggest that the MRSA retrieved from rodents and shrews are likely related to humans and livestock associated strains. Urban rodents have high carriage rate of MRSA, with similar antimicrobial-resistance patterns and molecular characteristics to those of human isolates. These results suggest that urban rodents may play as maintenance host or vectors for MRSA which is important to human health.

## Methods

### Ethics statement

This study’s protocol was approved by the Animal Ethics and Welfare Committee of the School of Public Health, Southern Medical University, Guangzhou, China. Animals were cared for according to the Rules for the Implementation of Laboratory Animal Medicine (1998) by the Ministry of Health, China. All surgical procedures were performed under diethyl ether anesthesia to minimize suffering. Endangered or protected species were not involved in this study.

The protocol of collecting MRSA isolates from patients was approved by the ethics committee of Southern Medical University, and was performed in accordance with the ethical standards noted in the 1964 Declaration of Helsinki and its later amendments.

### Sample collection

All samples were collected in a community of the Baiyun District of Guangzhou City, Southern China. Rodents and shrews were trapped on a single night each month from July 2015 to May 2016. The animals were trapped alive in iron cages (Yue-zong Co Ltd., Zhongshan, China). Trapped rodents and shrews were euthanized with diethyl ether and oropharynx swabs collected. During the operation, the trained personnel were protected from diethyl ether by wearing filtering facepiece respirators, safety chemical goggles, anti-static uniforms and chemical protective gloves. The swabs were soaked in 2 mL of 7.5% sodium-chloride broth (Land Bridge, Beijing, China) and incubated overnight at 37 °C for selective enrichment. Animal morphometric data were collected, including species (primary identification), sex, weight, body length (nose-to-rump), tail length, ear length, and sexual maturity. Subsequently, a full necropsy was performed. A small portion (~ 0.5 cm^3^) of the brain of each animal was retrieved and preserved in RNAlater® (Life Technologies, Grand Island, USA) and stored at − 80 °C for subsequent species identification by cytochrome B-gene sequencing [43, 44].

### Bacterial isolation and identification

To isolate *S. aureus*, the sodium-chloride broth was streaked onto mannitol-salt agar plates (MSA) (Land Bridge, Beijing, China) and incubated at 37 °C for 24 h. Presumptive *S. aureus* colonies, which appeared yellow on MSA plates, were sub-cultured onto fresh MSA plates and incubated at 37 °C for an additional 24 h.

Microbiological tests of the isolated colonies included oxidation and fermentation of MSA, the catalase test (BioMerieux, France), the hemolysin test (5% sheep blood agar), the coagulase test (Land Bridge, Beijing, China), and Gram staining. *S. aureus* (ATCC® 25923) was used as a reference for each test.

Bacterial genomic DNA from each isolate was extracted using a TIANamp Bacteria-DNA kit (Tiangen Biotech Co., Ltd., Beijing, China). The presumptive isolates were confirmed as *S. aureus* by polymerase-chain reaction (PCR) using the 16S rRNA gene and the *nuc* gene for identification [45]. The *mecA* gene was detected using PCR as described previously [45].

### Antibiotic susceptibility testing

*S. aureus* antibiotic susceptibility was conducted according to the guidelines of the Clinical and Laboratory Standards Institute (CLSI) [46]. A panel of 14 antimicrobials representing 13 different classes was used for susceptibility testing. The antibiotics were azithromycin, clindamycin, cefoxitin, penicillin, linezolid, rifampin, trimethoprim/sulfamethoxazole, minocycline, tetracycline, chloramphenicol, ciprofloxacin, gentamicin, nitrofurantoin, and vancomycin. The minimum-inhibitory concentrations (MICs) of *S. aureus* against vancomycin were determined by agar dilution as described previously [47]. Susceptibility to the remaining 13 agents was determined by the Kirby-Bauer disc-diffusion method [48]. *S. aureus* (ATCC® 29213) and *S. aureus* (ATCC® 25923) were used for quality control (QC). Results were interpreted according to CLSI guidelines. Cefoxitin-resistant isolates or *mecA*-positive isolates were identified as MRSA. Isolates that were not susceptible to ≥3 classes of antibiotics or were MRSA, were classified as multidrug-resistant *S. aureus* (MDRSA) [49, 50].

### Molecular characterization of MRSA isolates

*Spa* typing for MRSA isolates was conducted as described previously [51]. Results were analyzed with BioNumerics Software version 7.6 (Applied Math, Sint-Matens-Latem, Belgium).

Multilocus-Sequence Typing (MLST) analysis of 7 housekeeping genes was performed as described previously [52]. Sequence types (STs) were assigned using the MLST database (https://pubmlst.org/saureus/). The eBURST algorithm was used to assign individual STs to specific clonal-complex (CC) types. Clustering analysis was used to infer relationships among the isolates from different origins using BioNumerics Software version 7.6 (Applied Math, Sint-Matens-Latem, Belgium).

The PVL gene, encoding the Panton-Valentine Leukocidin toxin, and the human-specific immune evasion cluster (IEC) genes (including *scn*, *chp*, *sak*, *sep*, and *sea*) were detected by PCR as described previously [53, 54].

### MRSA isolates from humans

Eight MRSA isolates were obtained from hospital associated pneumonia inpatients in a teaching hospital, which was located within the rat-trapping region in the Baiyun District, were also assessed during the same period.

### Statistical analysis

Statistical analysis was carried out using SPSS 20.0 (IBM Corp., Armonk, NY, USA). The distributions of *S. aureus* and MRSA positive samples by animal species and season were compared using the Chi-Square test. A two-sided *p* < 0.05 was considered statistically significant.

## Data Availability

The datasets used and/or analysed during the current study available from the corresponding author on reasonable request.

## Abbreviations

AZM: Azithromycin
C: Chloramphenical
CA-MRSA: Community-associated infection
CC: Clonal-complex
CFX: Cefoxitin
CI: Confidence Interval
CIP: Ciprofloxacin
CLSI: Clinical and Laboratory Standards Institute
CM: Clindamycin
CN: Gentamicin
F: Nitrofurantoin
I: Intermediate
IEC: Immune evasion cluster
LA-MRSA: Livestock-associated MRSA
LZD: Linezolid
MDRSA: Multidrug-resistant *S. aureus*
MH: Minocycline
MICs: Minimum-inhibitory concentrations
MLST: Multilocus-Sequence Typing
MLVA: Multilocus variable-number tandem-repeat analysis
MRSA: Methicillin-resistant *Staphylococcus aureus*
MSA: Mannitol-salt agar plates
MSSA: Methicillin-susceptible *S. aureus*
NA: Not available
P: Penicillin
PA: Patient
PBP 2a: Penicillin-binding protein
PCR: Polymerase-chain reaction
PFGE: Pulsed-field gel electrophoresis
PVL: Panton-Valentine Leukocidin
QC: Quality control
R: Resistant
RD: Rifampin
RN: *Rattus norvegicus*
RR: Relative Risk
S: Susceptible
*S. aureus*: *Staphylococcus aureus*
SM: *Suncus murinus*
STs: Sequence types
SXT: Trimethoprim/sulfamethoxazole
TE: Tetracycline
VA: Vancomycin
VNTR: Variable-number tandem-repeat
